# Body Lice, *Yersinia pestis* Orientalis, and Black Death

**DOI:** 10.3201/eid1610.100683

**Published:** 2010-10

**Authors:** Mark Welford, Brian Bossak

**Affiliations:** Author affiliation: Georgia Southern University, Statesboro, Georgia, USA

**Keywords:** Black Death, plague, body lice, Yersinia pestis Orientalis, parasites, viruses, vector-borne infections, bacteria, letter

**To the Editor:** A scientific debate with public health implications wages: What caused the medieval European plague epidemics known as Black Death? Recent articles note inconsistencies between a rat flea–borne pandemic of *Yersinia pestis* (the bacterium that causes bubonic plague) and the documented characteristics of Black Death (*1*, among others). Ayyadurai et al. (*2*) acknowledge that a rat flea–only hypothesis does not fit Black Death observations, but they resolve theoretical transmission inconsistencies through a louse-borne hypothesis. Ayyadurai et al. base their surety of fact—that medieval “plagues” were caused by *Y. pestis* infection—on a 2007 study (*3*) in which 5 of 36 teeth of “plague” victims, none of which were dated to the Black Death era (1347–1351), contained biological evidence of *Y. pestis*. The 3 locations in that study were all port cities: 2 on the Mediterranean Sea and 1 on the Rhone River. As Duncan and Scott (*4*) note, bubonic plague most likely existed endemically near ship-borne trade, unlike the fast-moving epidemic fronts exhibited by medieval “plagues.” Moreover, Gilbert et al. (*5*) found no *Y. pestis* DNA in 61 skeletons from primarily nonport locations in England, France, and Denmark.

We do not dispute the authors’ claim that *Y. pestis* might have been present in some skeletons from port cities in France, or that body lice might, under certain circumstances, transmit the Orientalis biotype of *Y. pestis*; their work appears careful and considered. However, given the differences mentioned above and improved knowledge on the rapidity of virus mutation and worldwide transmission potential, we merely argue that the simplest explanation for medieval plagues has yet to be ruled out: that they may have resulted from a human-to-human transmitted virus. Adding complexity to an already complicated etiologic theory, and stating such as historical fact based on limited geography and sample size, does not seem congruent with Occam’s razor.
