# Supplementary material for: Body Lice, Yersinia pestis Orientalis, and Black Death
Source: Emerg Infect Dis. 2010 Oct;16(10):1649–51. doi: 10.3201/eid1610.100683 (PMC3294401; doi:10.3201/eid1610.100683)
Supplement: Related texts

# Reply: Body Lice, *Yersinia pestis* Orientalis, and Black Death

**Authors:** Robert G. McLean, Michael W. Fall

**Affiliations:** Author affiliation: National Wildlife Research Center, Fort Collins, Colorado, USA

**To the Editor:** The letter of Ayyadurai et al. (*1*) reminded us of a little-known paper (*2*) on rats and Black Death by our colleague and mentor David E. Davis. He researched and wrote in his retirement after years of research and reflection on rat ecology and rodent-borne diseases (*3**,**4*). *Rattus rattus* is commonly recognized as the vertebrate host of flea-borne plague that swept through Europe in the 1300s, killing >50% of the population. Davis believed this explanation did not fit what he knew of the ecologic requirements of fleas and black rats. He studied reports of archeologic excavations and reviewed poems, medieval bestiaries, and paintings and concluded that these rats were scarce during the Black Death era.

His theory, based on historical information and investigative trips to Europe, was that invasive rats, if present, mostly occurred in low densities in port areas, not in rural inland areas. He noted that the expected rodent die-offs with bubonic plague were not associated with human epidemics and that rodent fleas would not have been active during winter to transmit plague. Flea-borne transmission from rodents usually causes a few deaths per household, but deaths of entire households commonly occurred in the medieval epidemics. Human-to-human transmission of pneumonic plague must have occurred, but as described by Ayyadurai et al., there was evidence of human bubonic plague, suggesting vector involvement. Davis did not present a viable reservoir/vector hypothesis for plague transmission; this and the later, well-known association of *R. rattus* and other rodents with plague throughout the world, may partially explain why his ideas received little attention. The finding that human body lice can be bubonic plague vectors suggests a mechanism for human-to-human transmission continuing during winter in inland areas and, as suggested by the authors, could also explain total deaths in households.

## References

[1] Ayyadurai S, Sebbane F, Raoult D, Drancourt M. Body lice, *Yersinia pestis*, and Black Death. Emerg Infect Dis. 2010;16:892–3.2040940010.3201/eid1605.091280PMC2953993

[2] Davis DE. The scarcity of rats and the Black Death: an ecological history. J Interdiscip Hist. 1986;16:455–70. 10.2307/204499

[3] Anonymous. Profiles of previous Wildlife Disease Association leaders: David E. Davis 1913–1994. J Wildl Dis. 1995;31(suppl):15.7563419

[4] Davis DE. The characteristics of rat populations. Q Rev Biol. 1953;28:373–401. 10.1086/39986013121239

# Reply: Body Lice, *Yersinia pestis* Orientalis, and Black Death

**Authors:** Michel Drancourt, Didier Raoult

**Affiliations:** Author affiliations: Université de la Méditerranée, Marseille, France;; Editorial Board, Emerging Infectious Diseases, Centers for Disease Control and Prevention, Atlanta, Georgia, USA

**In Response:** Commenting on our recent demonstration that the human body louse was a likely vector of Black Death (the medieval European plague epidemics) (*1*), Welford and Bossak (*2*) point out that quantitative and qualitative inconsistencies in data for Black Death and modern plague argue against concluding that *Yersinia pestis* is the etiologic agent of Black Death (*3*). These authors acknowledge the paleomicrobiologic demonstration of *Y. pestis* in human remains collected at ports, yet they argue that such demonstration remains to be performed for human remains collected from inland burial sites (*2*).

Careful review of the literature indicates that 3 unrelated scientific teams have now demonstrated the presence of *Y. pestis*–specific biomolecules in 14th–18th-century human remains in 11 sites in Europe. These locations include 7 nonport, inland sites <650 km from the coasts (*4**–**6*) in addition to 3 Justinian (nonport) locations (*4**,**7*). Therefore, the fact that *Y. pestis* was the etiologic agent of Black Death can no longer be disputed; the inconsistencies correctly noted by Welford and Bossak actually question the reservoir and the vector of *Y. pestis* during the Black Death and the following epidemics rather than its cause. McLean and Fall remind us that the cumulative work of their mentor, David E. Davis, suggested that black rat ectoparasites could not have been likely vectors of medieval plague in Europe, based on the facts that expected die-offs of rats were not reported and that the rodents’ fleas would not have been active during winter in medieval Europe (*8*).

McLean and Fall acknowledge that our experimental data pave the way toward an alternative scenario of body louse–borne transmission of the Black Death. Such transmission of *Y. pestis* was observed by Blanc and Baltazard during a cluster of bubonic plague cases in households in Morocco during World War II (*9*). These authors demonstrated that the body louse could be infected when living on a septicemic patient, could stay alive for 7 days with infectious feces, and could transmit plague (*9*). Demonstration of *Y. pestis* in human lice collected from Black Death burials would be a step toward understanding the epidemiology of Black Death; this technically demanding approach has been successfully used to assess the transmission of typhus in soldiers of Napoleon’s Grand Army buried in Vilnius, Lithuania (*10*).

In agreement with these observations and those reported by Welford and Bossak (*3*), our work clearly indicates that *Y. pestis* could be efficiently transmitted by the human louse (*1*), a potential vector of Black Death because of its high prevalence in medieval Europe (*11*). Far from “adding complexity to an already complicated etiology theory,” as Welford and Bossak stated, it seems to us that the cumulative evidence provided by paleomicrobiologic demonstration and by our recent work (*1*) clarifies the epidemiology of the Black Death and the subsequent epidemics. Louse transmission of *Y. pestis* also explains inconsistencies rightly noted by Welford and Bossak and provides a reason for the current plague cases in poor areas of the world where poor hygiene is common. A search for alternative hypotheses, including the previous viral hypothesis for Black Death, may not be necessary (*2*).

The analogic reasoning based on observations of current infectious diseases cannot be applied to the medieval Black Death. Paleomicrobiologic evidence and historical data force us to change the paradigm and to question the established dogma about the epidemiology of plague. McLean and Fall remind us that, even in science, alternative hypotheses have trouble challenging dogma (*8*). Black Death is one of many areas at the intersection of microbiology and history for which many hypotheses have been proposed and none has received confirmation; these hypotheses have been repeated for so long that they became accepted as demonstrated truths.

## References

[1] Ayyadurai S, Sebbane F, Raoult D, Drancourt M. Body lice, *Yersinia pestis* Orientalis, and Black Death. Emerg Infect Dis. 2010;16:892–3.2040940010.3201/eid1605.091280PMC2953993

[2] Welford M, Bossak B. Body lice, *Yersinia pestis* Orientalis, and Black Death [letter]. Emerg Infect Dis. 2010;16:1651.2087530810.3201/eid1610.100683PMC3294401

[3] Welford MR, Bossak BH. Validation of inverse seasonal peak mortality in medieval plagues, including the Black Death, in comparison to modern *Yersinia pestis*–variant diseases. PLoS ONE. 2009;4:e8401. 10.1371/journal.pone.000840120027294PMC2791870

[4] Drancourt M, Roux V, Dang LV, Tran-Hung L, Castex D, Chenal-Francisque V, Genotyping, Orientalis-like *Yersinia pestis*, and plague pandemics. Emerg Infect Dis. 2004;10:1585–92.1549816010.3201/eid1009.030933PMC3320270

[5] Pusch CM, Rahalison L, Blin N, Nicholson GJ, Czarnetzki A. Yersinial F1 antigen and the cause of Black Death. Lancet Infect Dis. 2004;4:484–5. 10.1016/S1473-3099(04)01099-015288817

[6] Bianucci R, Rahalison L, Massa ER, Peluso A, Ferroglio E, Signoli M. Technical note: a rapid diagnostic test detects plague in ancient human remains: an example of the interaction between archeological and biological approaches (southeastern France, 16th–18th centuries). Am J Phys Anthropol. 2008;136:361–7. 10.1002/ajpa.2081818350578

[7] Wiechmann I, Grupe G. Detection of *Yersinia pestis* DNA in two early medieval skeletal finds from Aschheim (Upper Bavaria, 6th century AD). Am J Phys Anthropol. 2005;126:48–55. 10.1002/ajpa.1027615386257

[8] McLean RG, Fall MW. Body lice, *Yersinia pestis* Orientalis, and Black Death [letter]. Emerg Infect Dis. 2010;16:1651–2.20875309

[9] Blanc G, Baltazard M. Recherches expérimentales sur la peste. L’infection du pou de l’homme: *Pediculus corporis* de Geer. CR Acad Sci. 1941;213:849–51.

[10] Raoult D, Dutour O, Houhamdi L, Jankauskas R, Fournier PE, Ardagna Y, Evidence for louse-transmitted diseases in soldiers of Napoleon’s Grand Army in Vilnius. J Infect Dis. 2006;193:112–20. 10.1086/49853416323139

[11] Raoult D, Roux V. The body louse as a vector of reemerging human diseases. Clin Infect Dis. 1999;29:888–911. 10.1086/52045410589908
